# Asymmetric Silica–Gold Nano/Microparticles: Eccentric, Janus, Tadpole Structures, and Their Applications

**DOI:** 10.1021/acsami.6c02756

**Published:** 2026-04-13

**Authors:** Teagan Hamlett, Xiaowei Wang, Jaden Poellot, Joseph E. Doebler, Sid Hashemi, Xiao Li, Ying Bao

**Affiliations:** † Department of Chemistry, 1632 Western Washington University, Bellingham, Washington 98226, United States; ‡ Materials Science and Engineering Department, 3404 University of North Texas, Denton, Texas 76207, United States

**Keywords:** gold-silica nano/microparticle, ligand mediated, eccentricity, janus structure, tadpole structure, catalysis, liquid crystal

## Abstract

Asymmetric particles, characterized by asymmetries in composition, topology, or surface properties, have attracted increased attention due to their unique advantages, enabling versatile applications across a broad range of fields. Herein, we employ gold nanoparticles as cores and deposit silica to form a series of asymmetric structures, including eccentric, Janus, and tadpole morphologies. Polyacrylic acid (Mw: 1800 Da) and 4-mercaptophenylacetic acid are used for functionalizing the surface of gold nanoparticles prior to silica coating. We systematically investigate the role of ligands in directing silica shell formation and demonstrate that the position of the gold core within the silica shell can be precisely controlled by tuning the total ligand amount while maintaining a fixed ligand ratio. In addition, by adjusting the ligand ratio, a Janus structure can be obtained, which is then used as seeds for site-selective nucleation and growth of a second metal on the exposed Au surface, further breaking the structural symmetry. Through lowering the reaction pH from 10 to 8 and extending the aging time, tadpole structures with tails ranging from tens of nanometers to over one micrometer are fabricated. Finally, we briefly demonstrate potential applications of the asymmetric Janus and tadpole structures in a catalysis and liquid crystal system study.

## Graphical abstract

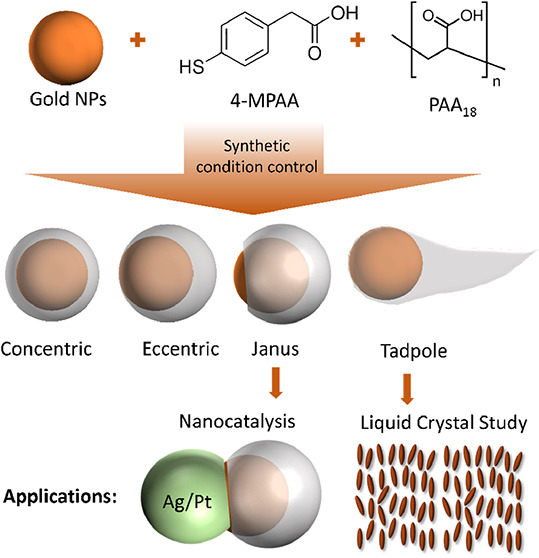

## Introduction

Asymmetric particles with different components or different surface physicochemical properties can contain several distinct properties simultaneously, such as hydrophilicity and hydrophobicity, optical properties, and magnetic properties, permitting versatile applications ranging from biosensing and drug delivery to energy conversion and catalysis.^1–3^ For example, compared to traditional core–shell symmetric structures, asymmetric particles with dual or multiple distinct domains such as heterodimers can have stronger synergic effects since the distinct domains can work or be manipulated/tailored independently without interfering with each other and even can cooperate with each other to realize significantly improved properties.^4–9^ Asymmetric particles having anisotropic shapes, such as tadpole or peanut shape, have demonstrated interesting effects by creating asymmetric topological defects with long-range distortion of director fields and subsequent distorted elastic forces in anisotropic liquids, such as liquid crystals (LCs) or active matters, in a more complex manner than effects from symmetric particles.^10,11^

It has been very attractive, though challenging, to develop approaches for fabricating asymmetric nano/microparticles. Until now, there have been numerous methods to design and fabricate such asymmetric particles, including bottom-up synthesis, template method, etching method, and so on.^12–15^ One of the most popular strategies to construct asymmetric particles is to utilize a presynthesized single-component particle as a core and overgrow secondary material on the surface of that core to create asymmetricity. Metal and silica are attractive paired components for such asymmetric particles. Metal nanoparticles such as gold and silver not only have prominent properties broadly used in many applications but also have extensively developed methods to obtain well-controlled size and morphology.^16,17^ Silica has often been used as the secondary material due to its advantages, including improving the stability of the nanoparticle, being relatively chemically inert, mechanical flexibility, and the effectiveness of attaching different functional groups to it.^18–20^ To date, a number of works demonstrate using asymmetric metal-SiO_2_ nanoparticles for various applications such as drug delivery,^21^ treatment of liver cancer cells,^22–24^ self-propelled motors,^25^ logic gates,^26^ and so on. The structures of asymmetric metal-SiO_2_ nanoparticles for those applications take a wide range, including eccentric/Janus,^25,27,28^ bullet,^24^ toothbrush,^22^ and so on.

One of the most popular strategies for creating asymmetry in nanoparticles is by using ligands to tune the surface wettability of their cores. The degree of surface wettability, closely related to the interfacial tension or surface energy, influences the interaction (affinity) between two components and guides secondary material deposition.^15,27,29^ Poly­(acrylic acid) (PAA) polymer paired with 4-mercaptophenylacetic acid (4-MPAA), 3-mercaptopropionic acid (MPA), or mercaptobenzoic acid (MBA) is often utilized to control the metal location and forms spherical silica shells on the resulting asymmetrical particles.^15,27,30^ To form more complex silica structures such as tadpole structures, the Kong group used CTAB in addition to PAA and 4-MPAA to tune the surface wettability between PAA and silica, as well as to form the CTAB/PAA aggregates as templates for guiding the silica shell growth into a hollow silica tadpole structure.^31^ Beyond that, there are rarely reports on accurate control of the location of the core metal nanoparticles inside the silica shell. Furthermore, forming asymmetric microstructures featuring microscale silica tails has never been previously reported, which could be of great interest to active matter scientists studying material in microscale.

In this work, we demonstrate that PAA influences silica formation and show that ligand composition can be used to tune the surface wettability of gold nanoparticles, thereby controlling the degree of asymmetry. We used as-synthesized citrate-stabilized gold nanospheres (GNPs) as cores and deposit silica shells to construct asymmetric structures including eccentric, Janus, and tadpole morphologies. PAA (PAA_18_, Mw: 1800 Da) and 4-MPAA were used for functionalizing the surface of gold nanoparticles prior to silica coating. The role of the ligands in directing silica shell formation is systematically investigated, and precise control over the location of the GNPs within the silica shell during encapsulation can be achieved by tuning the total ligand amount while maintaining a fixed ligand ratio. Furthermore, this mechanistic understanding enables the fabrication of an asymmetric Janus nanostructure, which is then used as seeds for site-selective nucleation and growth of a second metal on the exposed Au surface, further breaking the structural symmetry. This ligand-mediated control of surface wettability provides a rational design strategy for tuning particle eccentricity and achieving Janus structures, distinguishing our approach from conventional Janus fabrication methods. By tuning the reaction pH and aging time, we fabricated tadpole structures with tails ranging from tens of nanometers to over one micrometer. Finally, we briefly demonstrate the potential applications of the asymmetric Janus and tadpole structures in a catalysis and LC system study.

## Results and Discussion

### Formation of Asymmetrically Eccentric Nanostructures

To start, the citrate-coated GNPs were synthesized based on a modified method developed by Bastús et al.^32^ Figure S1a shows the morphology of the synthesized GNP. The image was obtained via scanning electron microscope with the transmission mode, which will be called a STEM image going forward. As presented in Figure S1b, the diameter of the synthesized gold nanospheres was 46.7 ± 3.9 nm. To coat with silica, the synthesized GNPs are coated and stabilized by citrate ions, which were added in an alcohol–water (2-propanol/water) cosolvent system. Silica was formed via hydrolysis and subsequent condensation of silica precursor (tetraethyl orthosilicate (TEOS)) at pH = 10 conditions (created by ammonia hydroxide). To manipulate the silica formation on the surface of GNP, PAA_18_ and 4-MPAA ligands were chosen to functionalize the surface of GNPs before silica shell formation. The carboxylic acid moieties from PAA_18_ polymer^33^ and the thiol group from 4-MPAA^34,35^ have a strong affinity on the surface of gold. Due to the high interfacial energy, 4-MPAA-coated GNP areas promote silica formation and condensation on the surface of gold, while PAA_18_-coated GNP areas do not.^36,37^ Therefore, when solely the 4-MPAA ligand was used in the system, nearly all GNPs were fully encapsulated by silica and formed concentric core–shell structures, as shown in Figure S2a. On the other hand, when solely PAA_18_ ligands are used, GNPs are not able to be encapsulated by silica, as shown in Figure S2b. To fabricate the asymmetrically eccentric nanostructure, a very low amount of PAA_18_ was introduced to mix with 4-MPAA (form [4-MPAA]/[PAA_18_] = 7.78) in order to functionalize the surface of GNP as shown in Figure 1a. The resulting silica shell fully encapsulates the GNP, while the nanoparticle has an eccentric core–shell structure; representative STEM images are shown in Figures 1b and S3. Note that not all nanoparticles appear to have eccentric formation, which is normal due to the various directions of orientation when nanoparticles are deposited on the substrate. From this result, it is clear that the introduced PAA_18_ ligands have affected silica deposition due to ligand competition.

**Figure 1:**
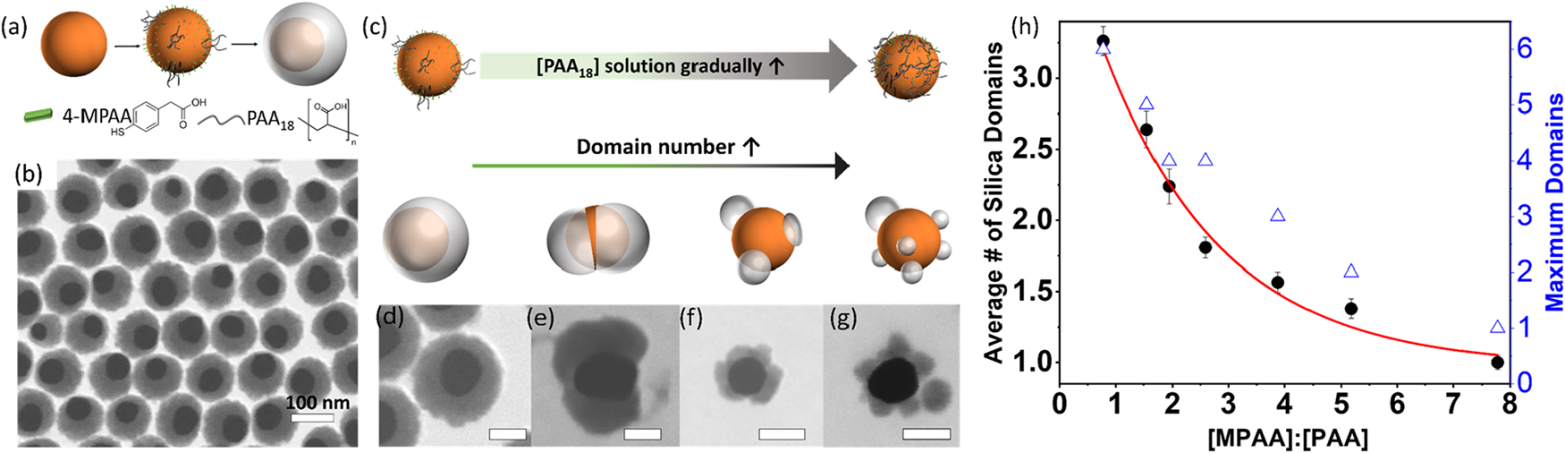
(a) Schematic diagram showing the formation of an eccentric silica shell on the gold nanosphere via ligand modification; (b) STEM image of silica-deposited GNPs using varied MPAA/PAA_18_ ratio with 7.78. (c) Schematic illustration of the concentration of PAA_18_ impacting the number of silica domains in the GNP. (d–g) STEM images of silica-deposited GNPs using varied MPAA/PAA_18_ ratio with (d) 7.78, (e) 5.17, (f) 1.94, and (g) 0.778. (h) Plot of the average number of silica domains versus the MPAA/PAA_18_ molar ratio, including the maximum domains of silica for each parameter. Scale bar: 50 nm.

#### Impact of the Amount of PAA_18_ in Solution

To better understand the ligand competition, we investigate the silica shell formation when gradually increasing the concentration of PAA_18_ solution, while the concentration of 4-MPAA in solution is maintained constant (molarity of 4-MPAA was 0.1059 mM). As described earlier, when a very low amount of PAA_18_ concentration was introduced ([4-MPAA]/[PAA_18_] = 7.78), the resulting silica-coated GNP nanoparticle was fully encapsulated by the silica shell, while the nanoparticle has an eccentric core–shell structure, as shown in Figures 1b,1d, S3, and S4a. When increasing the PAA_18_ concentration to reach the [4-MPAA]/[PAA_18_] = 5.17, the silica now partially coats the surface of GNPs and forms a maximum of two distinct domains, revealing one exposed area of GNPs to the external environment, as shown in Figures 1e and S4b. When increasing the concentration of PAA_18_ further ([4-MPAA]/[PAA_18_] = 1.94 and 0.778), the maximum number of silica domains on the GNPs increases from 3 in Figure 1f to a maximum of 6 in Figure 1e on nanoparticles, showing more distinct areas of exposed gold on the NP surface. The corresponding low-magnification images are in Figure S4c,d, respectively. Note that here we are focused on the number of formed domains instead of the size of these domains. This is because the size of the domain is irrelevant, as this is due to the reaction time. Since the STEM images used here can only view the silica coating on the side of the GNPs instead of a three-dimensional (3D) view of the resulting nanostructure due to the orientation of the deposited particle, some nanoparticles would appear fully coated even if they actually are partially coated. However, analyzing trends in the maximum and average numbers of silica domains on the GNPs for STEM images of all samples will still result in a meaningful conclusion, though the actual number of silica domains will be consistently biased downward. Figure 1h shows the plot of the average number and maximum number of silica domains on the GNPs as a function of the [4-MPAA]/[PAA_18_] ratio. The average number of silica domains was determined by measuring the maximum number of domains observed in each particle divided by the number of particles analyzed. Figure S4 shows examples of counting the maximum number of domains on each particle using orange crosses to indicate each domain. From the plot, it shows that as the ratio of [4-MPAA]/[PAA_18_] decreases, the average number of silica domains (or exposed areas of gold surface) increases exponentially, and eventually as the ratio approaches zero, the exposed area of gold surface is maximized since the silica is not able to deposit on the surface (as shown in the solely PAA_18_ ligand condition).

Based on the observed results, we believe that when using a mixture of 4-MPAA and a much lower concentration of PAA_18_, the ligands’ distribution on the nanoparticle surface was as shown in Figure 1a, step 2, where 4-MPAA ligands covered most of the GNP surface, while PAA_18_ ligands were sparsely distributed. The sparsely distributed PAA_18_ ligands decreased the overall wettability of GNPs generated by 4-MPAA. As a result, the silica would nucleate and grow on the eccentric core–shell structure, reducing the silica-gold interfacial area and thus enabling the minimization of interfacial energy. Similar phenomena were also reported by Zhao et al.^38^ In their work, they fabricated eccentric silica-gold nanoparticles using PVP to increase the wettability of the citrate-coated GNPs. With sufficient PVP ligands, the resulting structure is a concentric core–shell, while with fewer PVP ligands, the structure changes to an eccentric structure.^38^ As PAA_18_ ligands increase, the surface wettability decreases and, more importantly, with a high enough concentration of PAA_18_ ligands in the system, PAA_18_ ligands tend to form patches on the surface of GNPs, significantly decreasing the local wettability of GNPs. The 4-MPAA ligands cover the rest of the area for silica nucleate and form silica domains, while PAA_18_ patches disfavor the expansion growth of silica domains, resulting in discrete domains. The higher the concentration of PAA_18_, which results in a lower [4-MPAA]/[PAA_18_] ratio, as shown in Figure 1c–h, the more patches are formed, resulting in more silica domains. The formation of such patches under a mixture of different ligands when adsorbing on the surface has been shown in previous studies.^15,39–41^ It is worthwhile to note that the effect of ligand composition on surface wettability is inferred indirectly through systematic variation of 4-MPAA and PAA concentrations and the resulting changes in particle morphology and eccentricity. The observed trends in anisotropic growth are consistent with ligand-mediated modulation of the surface properties.

#### Impact of the Amount of 4-MPAA and PAA_18_ Mixture in Solution

Another strategy for studying competition when ligand-packing on the surface of GNPs is to adjust the molarity of 4-MPAA and PAA_18_, keeping their ratio fixed. By changing the molarity of both ligands in the solution while keeping the ligand molar ratio the same, the ratio of ligands to GNPs changes, impacting the ligands’ distribution due to the ligands’ affinity strengths to GNPs and thus strongly impacting the eccentricity of core–shell structures. The degree of eccentricity can be expressed by calculating the ratio of R1 to R2, as denoted in the schematic in Figure 2a. R1 is the diameter of the formed core–shell nanoparticle, and R2 is the distance from the center of the core to the nearest edge of the nanoparticle. If the core–shell nanoparticle is concentric, where the core is at the center of the nanoparticle, then the ratio of R1/R2 = 2. When the core–shell nanoparticle is eccentric, R1/R2 > 2.

**Figure 2:**
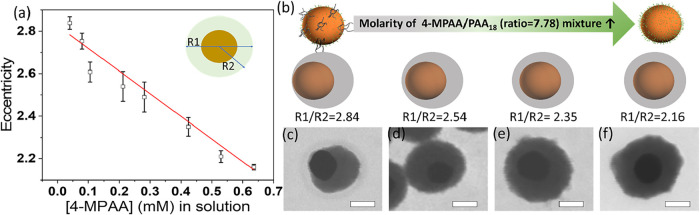
(a) Plot of the eccentricity of silica coating vs 4-MPAA ligand concentration in solution. (b) Schematic illustration of the molarity of 4-MPAA/PAA_18_ (ratio = 7.78) mixture impact on the eccentricity of silica coating on the GNP. (c–f) STEM images of silica coating on the GNP using varying concentrations of the 4-MPAA ligands with (c) 0.039 mM, (d) 0.21 mM, (e) 0.41 mM, and (f) 0.62 mM. Scale bar: 50 nm.

As shown earlier in Figure 1b, when 16 μL of 4-MPAA and PAA_18_ with [4-MPAA]/[PAA_18_] = 7.78 was used in the synthesis condition (molarity of 4-MPAA was 0.1059 mM), the resulting nanoparticles have R1/R2 = 2.61, indicating an eccentric core–shell nanostructure. Keeping the ratio of [4-MPAA]/[PAA_18_] = 7.78 constant, the molar concentrations of 4-MPAA and PAA_18_ were increased or decreased to study the effects on ligand competition and the resulting eccentricity of the spherical nanostructures. The relationship of the eccentricity of the resulting nanostructures versus the corresponding molarity of 4-MPAA in solution was found and is shown in Figure 2a. Note that the ratio of [4-MPAA]/[PAA_18_] = 7.78 remained constant throughout; therefore, the molarities for both ligands were changing simultaneously, even though only the molarity of 4-MPAA is plotted in this figure. When decreasing [4-MPAA] from 0.1059 to 0.0795 mM (with a corresponding decrease in PAA_18_), the position of the core within the structure shifted significantly away from the center, with R1/R2 changing from 2.61 to 2.75. In contrast, when [4-MPAA] increased from 0.1059 to 0.6356 mM (with a corresponding increase in PAA_18_), the position of the core shifted toward the center, and R1/R2 changed from 2.61 to 2.16. Figure 2b is a schematic illustration showing the position of the core gradually moving from the side of the structure to the center. Figure 2c–f shows representative STEM images of nanostructures, and the corresponding low-magnification images are in Figure S5.

From these observed results, we believe that when both ligand molarities are low, both 4-MPAA and PAA_18_ can cover GNPs, resulting in a surface with reduced wettability and an eccentric structure. However, with very high ligand molarities, 4-MPAA covered nearly all of the GNPs, while PAA_18_ barely covered any GNPs, resulting in fully wetting the GNPs and thus a concentric structure. This is not surprising since the thiol group in 4-MPAA has a strong binding affinity to the GNPs (thiol-gold interaction), while the PAA_18_ ligands have a much weaker electrostatic interaction. By increasing the concentration of both ligands in solution, we can see the growth trend toward the morphology of growth made solely with MPAA, showing that MPAA is outcompeting the PAA_18_. Overall, accurate control of the location of the core NPs inside the shell during encapsulation can be achieved, demonstrating that this method can quantitatively control the degree of eccentricity at the nanoscale.

### Formation of Asymmetric Janus Nanostructure

We investigated the roles of PAA and 4-MPAA and their competitive interactions in our system. Our results show that increasing the PAA content reduces the effective wettability of the gold nanoparticle surface, directing anisotropic growth and enabling controlled evolution from eccentric structures to Janus-like structures under highly eccentric growth conditions. Further decreasing the surface wettability by reducing the 4-MPAA concentration below 0.0795 mM while increasing the PAA_18_ concentration (i.e., lowering the [4-MPAA]/[PAA_18_] ratio) leads to increased eccentricity of the resulting nanostructures and ultimately yields Janus nanostructures. In general, “Janus nanostructures” refers to particles possessing dual surface functionalities or consisting of two jointed components with distinct properties.^42,43^ Figure 3a shows a Au-SiO_2_ Janus nanostructure obtained using [4-MPAA]/[PAA_18_] = 2.59 with 0.039 mM 4-MPAA concentration; details are found in the Experimental Section. A certain amount of the nanostructures have exposed gold surfaces, and thus, these structures possess gold and silica dual surfaces. The same synthesis parameters were applied to 30 nm GNP (as compared to the 50 nm default size) to form Janus nanostructures, with a representative STEM image shown in Figure 3b. Compared to the 50 nm GNP-based Janus nanostructure, the 30 nm GNP-based Janus nanostructures have a greater share of their gold core exposed to the environment. Larger area STEM images for those two nanostructures are found in Figure S6. It is worth noting that since STEM provides a two-dimensional projection, Janus particles can appear fully encapsulated, depending on their orientation on the grid. Therefore, the two-dimensional (2D) visual appearance in the selected images does not necessarily fully capture the true distribution of structures.

**Figure 3:**
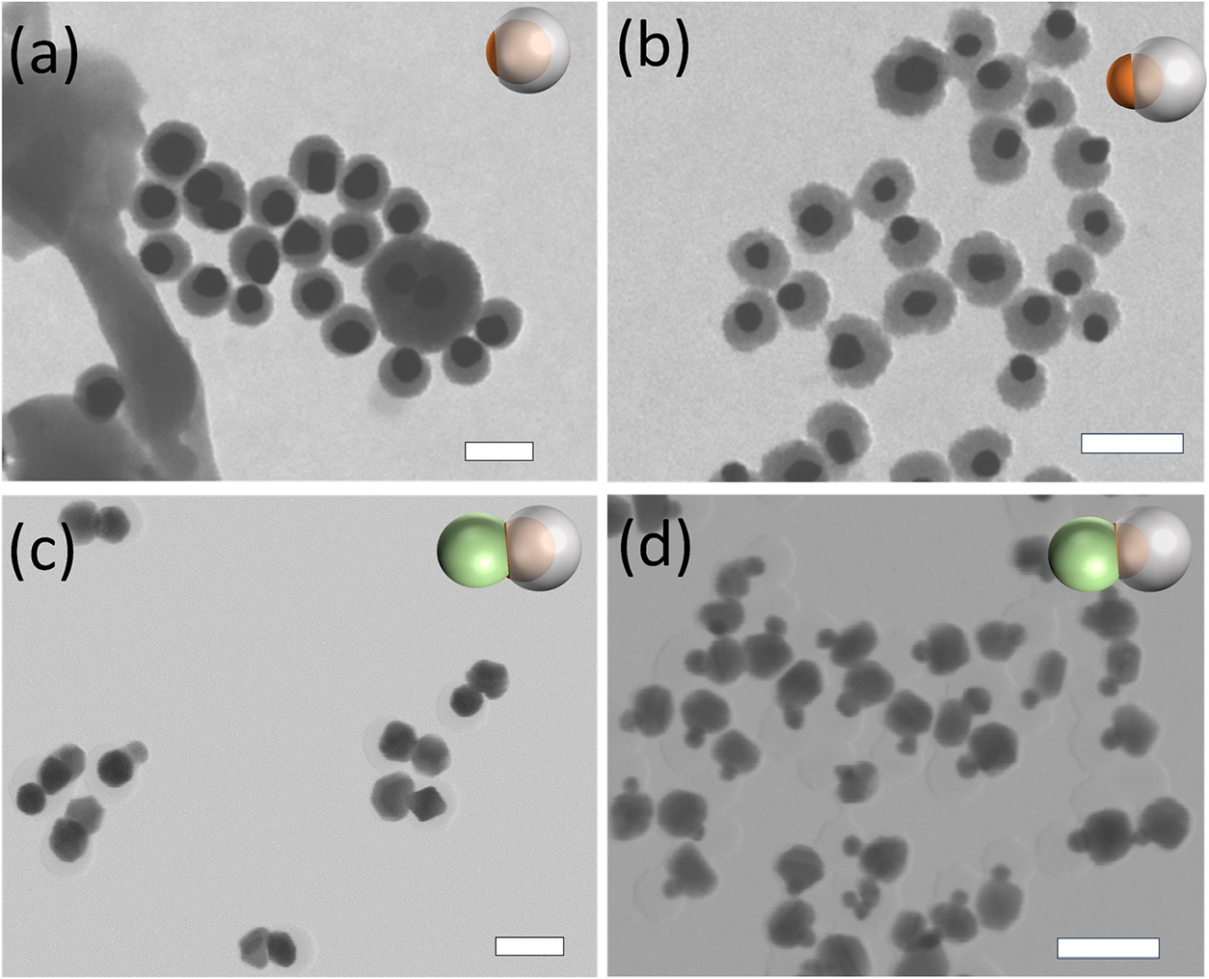
(a, b) STEM images of Au-SiO_2_ Janus nanostructures using [4-MPAA]/[PAA_18_] = 2.59, while 0.039 mM 4-MPAA concentration using GNP core size with (a) 50 nm and (b) 30 nm. (c, d) STEM images of ternary Ag–Au–SiO_2_ nanostructures after Ag depositing on Au-SiO_2_ Janus nanostructures. (c) 50 nm GNP core size and (d) 30 nm GNP core size. Scale bar: 100 nm.

#### Second Metal Deposition

To both better evaluate the fraction of Janus nanostructures formed and demonstrate the utilization of the unique Janus nanostructure, the synthesized Janus nanoparticles were further used as seeds for site-selective nucleation and growth of a second metal on the exposed Au surface, further breaking the structural symmetry. In our study, silver is used as the second metal to grow on the exposed surface, forming a ternary Ag–Au–SiO_2_ nanostructure (also called heterotrimer structures). The typical growth solution was prepared by adding hydroquinone (HQ) and AgNO_3_ into polyvinylpyrrolidone (PVP) solution (details are found in the Experimental Section). Figure 3c shows typical images of Ag selectively grown from the exposed Au surface on a 50 nm GNP-based Janus nanostructure (Ag–Au–SiO_2_ nanostructure), which has a yield of ∼75% and is reasonably uniform. Due to the intrinsic lower contrast of Ag relative to Au under the electron beam, the heterostructure can be easily identified. Figure 3d has typical images of Ag selectively grown from the exposed Au surface on a 30 nm GNP-based Janus nanostructure, which has a much higher yield (∼90%). This shows that 30 nm GNPs produce a much higher percentage of Janus nanostructures compared to 50 nm GNPs, indicating that the 30 nm GNPs have lower wettability under the same ligand conditions. To examine the ternary Ag–Au–SiO_2_ nanostructure resulting from the Janus structure of silica shell on GNP, we employed the same silver reduction process using the purified GNPs as seeds, and the resulting nanoparticles show Ag fully coating the Au core, forming symmetric core–shell nanostructures instead of the ternary Ag–Au–SiO_2_ nanostructure (Figure S7). This indicates that the Janus silica shell is necessary to facilitate the generation of the heterotrimer Ag–Au–SiO_2_ nanostructure. We also characterized both the Au-SiO_2_ Janus nanostructure and the ternary Ag–Au–SiO_2_ nanostructure by energy-dispersive X-ray (EDX) spectroscopy mapping analyses (Figure 4). The overlay image in Figure 4a confirms the asymmetric Janus nanostructure of the Au-Silica formation. The silver element image in Figure 4b shows the Ag present in the ternary Ag–Au–SiO_2_ nanostructure, and the overlay image shows the asymmetric coating of Ag on Au, forming the heterostructure.

**Figure 4:**
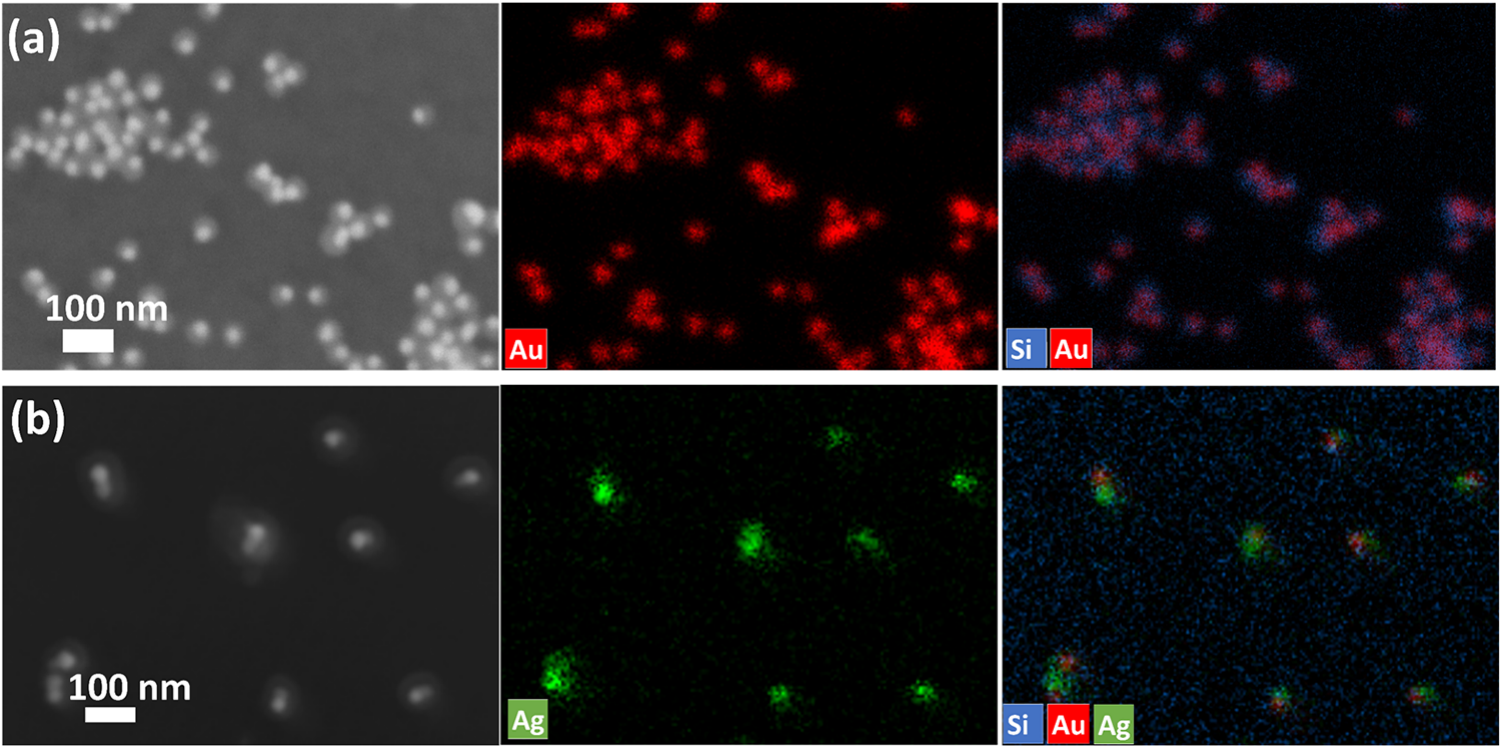
SEM (left) and elemental mapping images (right) of (a) Au-SiO_2_ Janus nanostructure and (b) ternary Ag–Au–SiO_2_ nanostructure. In the elemental mapping image, the red, blue, and green colors represent Au, Si, and Ag atoms, respectively.

Via a galvanic replacement reaction (GRR) between silver and K_2_PtCl_4_ precursor, the silver domains on the as-obtained Ag–Au–SiO_2_ nanostructure act as sacrificial templates and are fully or partially replaced by platinum (Pt) to form a hollow nanostructure.^44,45^ This process results in an asymmetric hollow heterotrimer incorporating Pt. Due to higher yield, the heterotrimer Ag–Au–SiO_2_ nanostructure with 30 nm gold core was used for the Pt formation, which is performed by simply titrating K_2_PtCl_4_ precursor into the Ag–Au–SiO_2_ nanostructure solution at room temperature. The color of the solution changes from grayish orange to dark gray, indicating the formation of Pt. Figure 5a shows that following the GRR process, the silver domain in heterotrimer nanostructure is replaced by Pt, resulting in a hollow structure, while the gold core remains intact (Pt/Ag–Au–SiO_2_ nanostructure). Yellow arrows in Figure 5a,5d show some examples of the intact gold cores.

**Figure 5:**
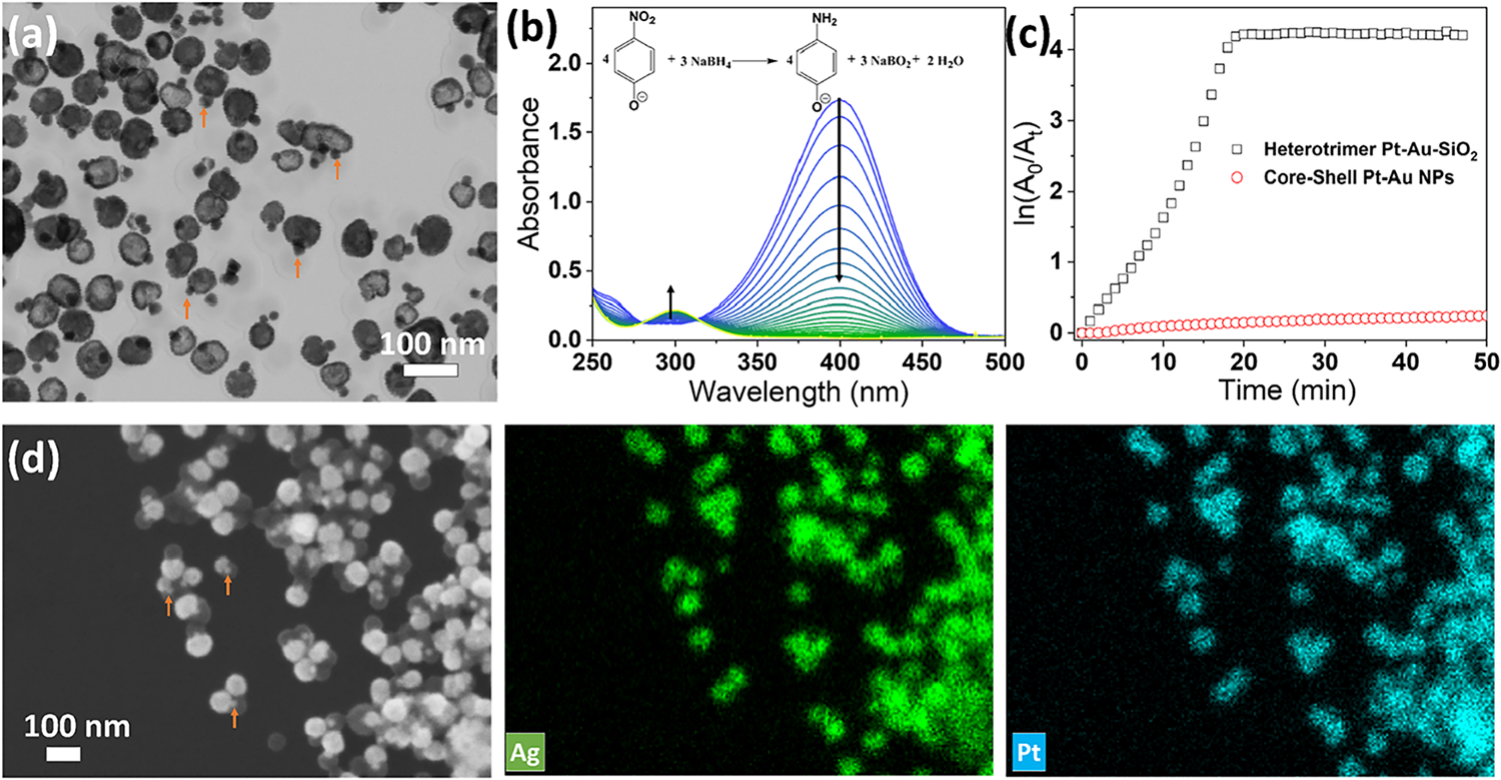
(a) STEM image of Pt/Ag–Au–SiO_2_ nanostructures obtained via GRR between silver and K_2_PtCl_4_ precursor; (b) time-dependent UV–vis absorption spectra of 4-NP reduced by NaBH_4_ and catalyzed by the obtained Pt/Ag–Au–SiO_2_ nanostructures; (c) graph of ln­(A_0_/A_t_) as a function of reaction time giving the comparative rate constants of Pt/Ag–Au–SiO_2_ nanostructures and Pt–Au core–shell nanostructures; and (d) SEM (left) and elemental mapping images (right) of Pt/Ag–Au–SiO_2_ nanostructure. In the elemental mapping image, the green and cyan colors represent Ag and Pt atoms, respectively. Yellow arrows show some representative intact GNPs in the nanostructures.

#### Catalysis Evaluation

Here, we used the catalytic reduction of 4-nitrophenol (4-NP) as a model system to study the catalytic properties of the obtained Pt-based heterodimer nanostructure. In a typical experiment, the 4-NP solution was first mixed with NaBH_4_ solution. Although the concentration of NaBH_4_ is much higher than that of 4-NP, no reaction could be observed in the absence of catalysis. When these nanostructures were added to the reaction system, the yellow solution became colorless gradually, implying the reduction of 4-NP. The reaction process was monitored by collecting the UV–visible absorption spectrum of the mixture of 4-NP and NaBH_4_ at different times. As shown in Figure 5b, initially, the solution showed an absorption peak at ∼400 nm due to the formation of 4-nitrophenolate ions, caused by the addition of NaBH_4_ solution. With the addition of the catalyst, the absorption peak at ∼400 nm decreases, and a new peak at 315 nm associated with -NH_2_ of 4-aminophenol (4-AP) appeared and increased with reaction time, demonstrating that the 4-NP has been rapidly and effectively reduced to 4-AP under the acceleration of catalysts. The kinetic reaction rate (k) could be evaluated by a pseudo-first-order kinetics, which could be estimated from the plot of ln­(A_0_/A_t_) versus the reaction time, in which A is the absorption at 400 nm, while A_0_ and A_t_ are the absorbance at the initial stage and successive intervals, respectively. As plotted in Figure 5c, the reaction is complete after about 18 min, suggesting a high catalytic activity of the nanostructures. As a comparison, the core–shell Pt–Au NPs, fabricated by directly depositing Pt on the surface of purified GNP (the morphology is shown in Figure S8a), have also been used to catalyze the reduction process. After adding the nanoparticles, no color change was observed for over an hour. Figure S8b shows the collected UV–visible absorption spectra over time, as well as the plot of ln­(A_0_/A_t_) as a function of reaction time in Figure 5c, noting that the reaction process has not effectively progressed.

To reveal the composition of the hollow structure after GRR, EDX mapping was used, and the results are shown in Figure 5d. Three components of the structures are clearly shown in the scanning electron microscope (SEM) image. The Pt/Ag component and GNP are the brightest due to their high atomic number. The GNP is much smaller in size, as indicated by the arrow. The silica shell is the most transparent component due to having the lowest atomic number. The EDX mapping result reveals that after GRR, the silver domain results in the Pt/Ag alloy.

Three factors could contribute to the higher catalytic efficiency from Pt/Ag–Au–SiO_2_ nanostructures as compared with core–shell Pt–Au NPs. First, traditional hollow structures have shown good prospects in the catalytic field by virtue of their large surface area and low density.^46^ The synthesized particles possess a hollow structure with an overall diameter of ∼40 nm, which provides a larger accessible surface area compared to the core–shell Pt@Au particles (∼35 nm total diameter with an ∼5 nm Pt shell). This structural difference likely increases the number of available active sites. Second, the plasmonic enhanced catalysis effect of bimetallic Pt/Ag alloy may contribute to enhanced catalytic performance as compared to monometallic Pt in the core–shell structure, which has also been reported.^47^ Finally, we believe that the spatial arrangement of the Au and Pt/Ag alloy domains in our heterotrimer nanostructure can significantly influence the catalytic performance. Majima et al. reported that bimetallic heterostructures with Pt nanoparticles grown along the edges of gold triangular nanoprisms exhibit 3–5 times higher catalytic activity than the counterpart heterostructures with Pt nanoparticles grown on the tips and randomly covering the entire surface of gold nanoprisms.^48^ The detailed mechanism of the heterotrimer contribution will be of interest to future design of catalysis material and should be studied further, which we will explore in the near future.

### Formation of Asymmetric Tadpole Nano/Microstructure

To further develop asymmetric nanostructure formation, we investigated the impact of pH on the formation of silica shell under synthesis conditions for Figure 1b (16 μL of 4-MPAA and PAA_18_ with [4-MPAA]/[PAA_18_] = 7.78 were used in the synthesis conditions (molarity of 4-MPAA was 0.1059 mM)). The reaction rate of TEOS hydrolysis and condensation, which is silicate oligomerization in the Stöber method, depends on the solution pH. In general, an increase in pH brings an increase in the hydrolysis rate, which produces higher concentrations of silane monomer.^34,49,50^ It has been reported experimentally and theoretically that the condensation rate peaks at pH 8, with an increase or decrease of pH from that point, reducing the condensation rate.^51,52^ To this point, all of the silica shells above were all formed under pH 10 conditions, and the formed silica shells from the core–shell structure have dense and spherical morphologies. As shown in Figure 1b, the silica shell formed at pH 10 has dense and spherical morphology. When the pH decreases to 8, instead of forming dense and spherical morphologies, silica monomers condense into tail structures, forming the tadpole structure (as shown in Figure S9). This is an interesting finding since there is no surfactant in the solution to act as the tail template, which has recently been reported to help the formation of tail structures.^53^ The formation of the tail is likely due to the change in kinetic balance between TEOS hydrolysis and subsequent condensation. When the silicane monomer supplies decrease, the area where the siloxane network has already formed on the surface of GNPs will continue to consume more monomer, which depletes the monomer available for condensation in other areas. Thus, the siloxane network will form asymmetrically. It is also worthwhile to note that similar tadpole structures can be formed by using various-sized GNP, as we tried using 50, 70, and 100 nm-sized GNPs. When the pH decreases to 7, TEOS hydrolysis and condensation are very slow and often incomplete, which results in no silica shell at all, as shown in Figure S10. More interestingly, the tail can grow to a much longer length based on the aging time. Figure 6 (using 70 nm GNPs) shows the tail length of about 45 nm after aging for 24 h and 300 nm after aging for 48 h. After aging for 120 h, the tail length continues to grow as long as ∼1500 nm. It should be noted that the tail length no longer increases after 120 h. We note that phase-separated or droplet-like domains induced by salt effects have been reported in related systems.^53–55^ The formed tadpole structure shown in Figures 6b,C, and S9 reveals open-ended and less dense structures. Thus, to form a long tail, the phase-separated or droplet-like domains may play a significant role here. In our system, the PAA–NH_4_^+^ complex may exhibit limited solubility in the alcohol-rich environment, which could potentially lead to localized liquid-like domains. While we do not have direct experimental evidence for droplet formation in the present study, such a mechanism is consistent with previously reported salt-induced phase separation and may contribute to the observed tail growth. However, unlike other related systems, their formed long tails are hollow capsule structures, which is not observed in our system; thus, liquid droplets have a less significant impact in our system than theirs. Additional efforts to more fully understand this mechanism are planned for future work.

**Figure 6:**
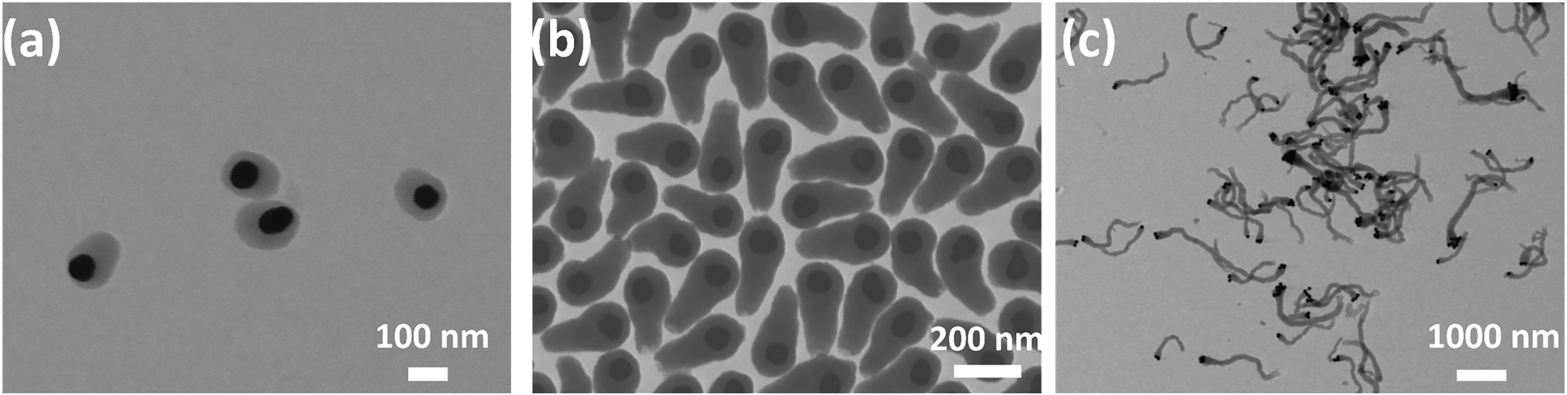
Representative STEM images of asymmetric tadpole structure with aging time of (a) 24 h, (b) 48 h, and (c) 120 h.

#### Tadpole Structure in LC Study

LCs are a unique class of soft materials characterized by anisotropic molecular orientation combined with fluidic properties.^56–61^ The shape, symmetry, and topology of colloidal particles provide powerful handles to tune colloidal phase behavior and physical properties of the LC system, enabling both fundamental physics studies and the design of functional materials.^62–64^ The asymmetric tadpole nano/microstructures allow for the exploration of the influence of asymmetry-driven morphology on the formation of topological defects in nematic LCs. Our recent paper^65^ has revealed that asymmetric features of particles could create abnormal “butterfly” defects in nematic LCs, and the consistent fluctuation from the flexible SiO_2_ tail could be coupled with elastic distortion to create larger magnitude cavities in anisotropic liquids. Following the same mechanism we used previously in the tadpole particle-nematic LC system, here, we provide a detailed observation on how the length of the tail affects the morphology of topological defects and the formation of cavitation in the nematic LC system.

We dispersed tadpole particles of the same GNP sphere head (core) size but with different tail lengths into 4-cyano-4′-pentylbiphenyl (5CB) nematic LCs and injected the mixture solution into an LC cell, as depicted in Figure 7a. The cell consisted of a bottom silicon substrate coated with hydroxyl-terminated poly­(6-(4-methoxy azobenzene-4′-oxy)­hexyl methacrylate (PMMAZO)), promoting tangential anchoring of mesogens at the particle surface, and a top glass slide-coated with octadecyltrichlorosilane (OTS), inducing perpendicular anchoring. This dual-anchoring configuration establishes a controlled environment for observing distinct morphological responses.

**Figure 7:**
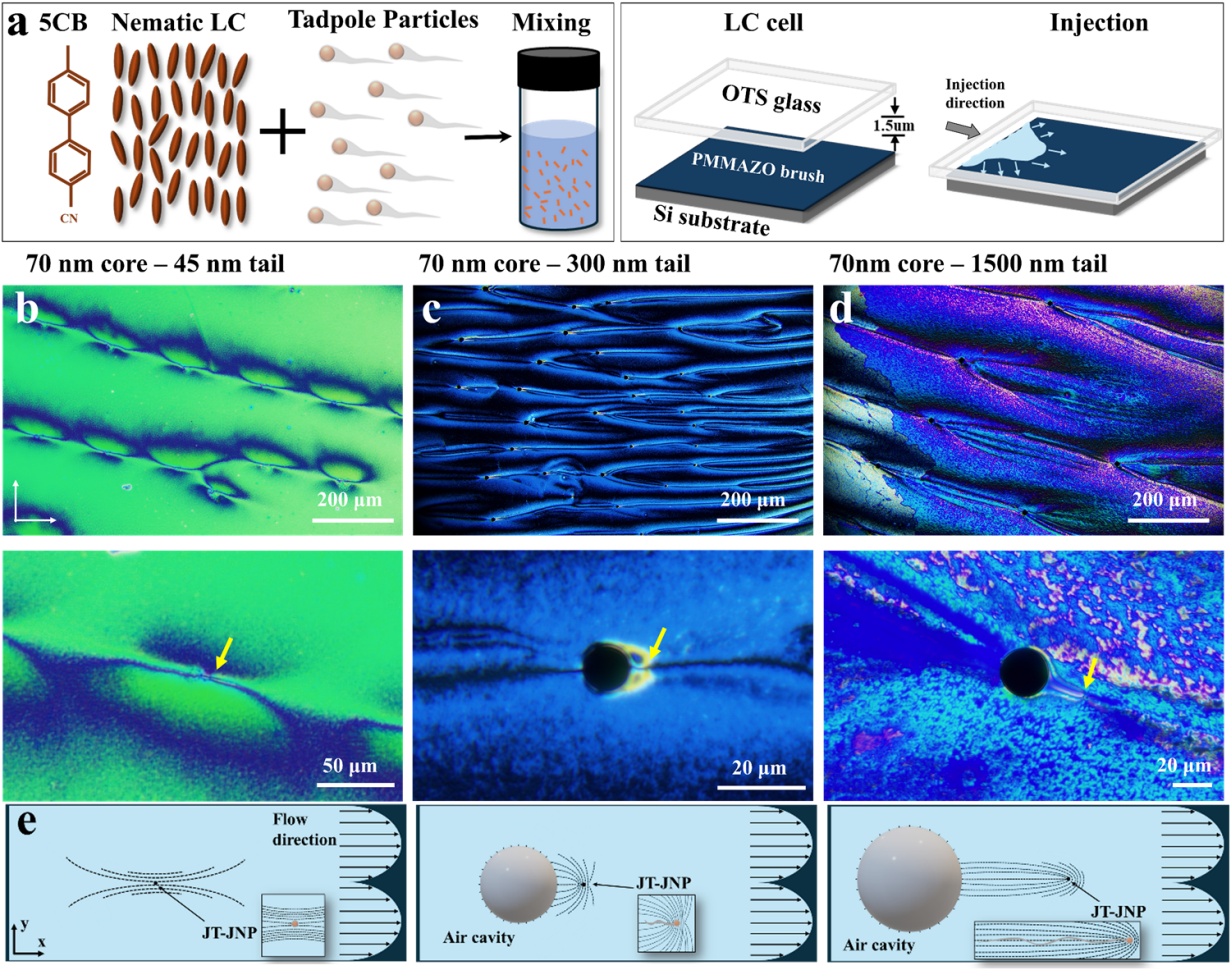
Surface morphology of tadpole particles confined in an LC cell with a Si substrate modified by PMMAZO surface anchoring. (a) Schematic of the solution preparation and LC cell assembly. Tadpole particles with a fixed core diameter of 70 nm and varying tail lengths are shown: (b) 45 nm, (c) 300 nm, and (d) 1500 nm. Their location is indicated with a yellow arrow in the bottom images of (b–d). (e) Schematic illustration depicting the deformation of the surrounding LC host and the formation of associated cavities induced by tadpole particles. The short black lines at the cavity boundary indicate homeotropic anchoring at the air interface.

Figure 7b–d highlights the various topological defect morphologies in LCs induced by tadpole particles with differing tail lengths. In Figure 7b, a tadpole particle with a short tail (70 nm core–45 nm tail) appears as a small dark nucleus encircled by a faint concentric ring, reflecting minimal director distortion that was insufficient to create an observable cavity. In contrast, extending the tail length to 300 nm (70 nm core–300 nm tail) significantly altered the interaction between tadpole particles and the LC matrix, as illustrated in Figure 7c, where distinct cavities emerged, marked by a dark circular region formed due to the particle’s motion. The particle’s elongated tail created a visible directional perturbation as a line defect, indicating a local discontinuity in the nematic director order. These cavities exhibit an average diameter of approximately 17 μm, with an average particle (indicated by the yellow arrow)-to-cavity distance of around 6 μm, clearly demonstrating how moderate tail lengths induce substantial, measurable volumetric responses. When the tail length is further increased to 1500 nm (70 nm core–1500 nm tail), as shown in Figure 7d, the system exhibits enhanced complexity. The longer tail intensifies elastic deformation and promotes irregular defect configurations, resulting in cavities with an average diameter of approximately 23 μm and an increasing particle (indicated by the yellow arrow)-to-cavity distance of approximately 25 μm. The formation of irregular defect structures and associated cavitation can be interpreted as a strongly coupled elastic–hydrodynamic response induced by the geometric asymmetry of the tadpole particle. The elongated tail introduces a pronounced anisotropic distortion in the surrounding nematic director field, leading to the development of extended disclination lines that propagate preferentially along the tail axis rather than remain localized near the particle core. Our recent study has demonstrated that similar asymmetric tadpole particles can induce strong director distortions and flow perturbations in nematic systems, leading to the formation of air cavities.^65^ Building on this understanding, as the particle moves through the LC under a capillary-driven flow, these asymmetric distortions continuously redistribute elastic stress and generate spatial gradients in the director orientation. The flexibility of the tail further amplifies these distortions by dynamic fluctuation, which enhances local flow perturbations and produces a directional wake behind the particle. Within this region, the combination of elastic deformation and flow-induced velocity gradients gives rise to a transient decrease in local pressure. Once this pressure drop exceeds a critical threshold, air is entrained, and a cavity nucleates behind the particle. Importantly, the cavity remains coupled to the particle through the surrounding distorted director field, resulting in a continuous defect pathway that links the particle and the cavity. This coupling explains the observed correlation between tail length, defect complexity, and cavity size, as longer tails generate more extensive distortions and sustain a stronger nonequilibrium pressure field, thereby promoting the formation of larger and more stable cavities compared to shorter or symmetric particles.

Figure 7e further shows the evolution of defect-line configurations by linking the particle geometry to flow-induced director deformation. For the short-tail particle (45 nm tail length), the distortion remains confined in the immediate vicinity of the particle, and the dashed defect lines retain a nearly symmetric distribution, indicating that the induced perturbation is too weak to propagate into the surrounding LC. As the tail length increases to the intermediate regime (300 nm tail length), this balance is disrupted, and the defect lines begin to extend preferentially along the flow direction, reflecting a transition from localized to directionally biased deformation. This change suggests that the elongated geometry introduces an inherent asymmetry that couples more effectively with the imposed flow. When the tail is further increased (1500 nm tail length), the defect lines become highly stretched and continuously form an extended deformation pathway that links the particle to the downstream region. Such a configuration indicates that the long tail sustains and amplifies the distortion over a larger spatial scale, reinforcing the observed increase in cavity size and separation. These observations also indicate that the formation of LC–air cavities in this system is predominantly governed by nonequilibrium kinetically driven processes. During injection, the imposed flow introduces transient stresses that disrupt the local director field, particularly in regions where asymmetry enhances spatial gradients in orientation. These dynamic perturbations facilitate local instabilities that enable cavity nucleation and growth. The absence of such features in the short-tail case further suggests that cavitation does not arise from an equilibrium defect configuration but instead requires sufficient disturbance to overcome local elastic resistance, showing the essential role of flow-induced dynamics.

Based on the above three types of tadpole particles with the same spherical head diameter (70 nm) but distinct tail lengths: 45 nm (short), 300 nm (medium), and 1500 nm (long), how the changes in tail length affect defect structures and cavitation in nematic LCs has been determined. By systematically varying only the tail length while keeping all other parameters constant, we found that increasing the tail length can alter the spatial arrangement of the director field and the defect morphologies, including the formation and characteristics of LC–air cavities. Specifically, our results reveal that longer tails amplify elastic deformation, enhance defect complexity, and promote larger and more distant cavitation events, thus directly linking tail-induced asymmetry to both defect structure and cavitation behavior in the nematic matrix. These findings confirm that the asymmetry-driven morphology of tadpole particles plays a central role in modulating the local LC order, defect structures, and cavitation phenomena. Furthermore, the ability to control and understand these effects using model asymmetric particles highlights their potential utility for studying shape-dependent phenomena in soft matter systems and for mimicking active matter behavior, such as targeted cargo transport, drug delivery, and creating responsive LC-based sensors or designing advanced adaptive materials.

## Conclusion

In summary, various asymmetric structures, including eccentric, Janus, and tadpole morphologies, were synthesized by using as-synthesized citrate-stabilized GNPs as cores and then depositing silica in a controlled manner via ligands, pH, and aging time. The impact of 4-MPAA and PAA_18_ ligands on the GNP surface and the resulting silica shell formation and eccentricity was explored and studied in detail. With a mechanistic understanding of eccentricity control, the asymmetric Janus structure was fabricated by lowering the ratio of [4-MPAA]/[PAA_18_] to further decrease surface wettability. The Janus nanostructures were then used as seeds for site-selective nucleation and growth of a second metal on the exposed Au surface, further breaking structural symmetry. Through controlling the reaction pH and aging time, tadpole structures with tails ranging from tens of nanometers to over one micrometer were fabricated. Finally, we briefly demonstrated the potential applications of the asymmetric Janus and tadpole structures in both catalysis and a LC system.

## Experimental Section

### Chemical and Materials

Tetrachloroauric acid trihydrate (HAuCl_4_·3H_2_O, ≥ 99.9%), 2-propanol anhydrous, 4-mercaptophenylacetic acid (4-MPAA, 97%), poly­(acrylic acid) (PAA_18_, average Mw = 1,800), and tetraethyl orthosilicate (TEOS) (99.9%) were purchased from Sigma-Aldrich. Sodium citrate dihydrate (99.0–101.0%) was purchased from Fischer Scientific, ethanol was purchased from Pharmco, and ammonium hydroxide was purchased from Macron Fine Chemicals. The nematic liquid crystal 4-cyano-4′-pentylbiphenyl (5CB) was purchased from Grand Winton Inc. Octadecyltrichlorosilane (OTS) was obtained from Sigma-Aldrich. The azobenzene-containing polymer PMMAZO, poly­(6-(4-methoxy-azobenzene-4′-oxy)­hexyl methacrylate), was synthesized following the procedure reported by Stewart and Imrie. Nanopure water with a resistivity of 18 MΩ cm was used in all experiments. All chemicals were used as received without further purification.

### Synthesis of Gold Sphere Seeds (∼17 nm)

To prepare the gold seeds, 150 mL of 2.2 mM sodium citrate solution was heated in a three-neck round-bottom flask under vigorous stirring until boiling. Then, 1 mL of 25 mM HAuCl_4_ was added, and the solution was left at reflux for 10 min before it was cooled to 90 °C. The initially clear solution changed to a transparent pink upon the addition of the gold and deepened in color until it was a wine red. Once the mixture was at 90 °C, 1 mL of 25 mM HAuCl_4_ solution was added. After 30 min, 1 mL of 25 mM HAuCl_4_ solution was added again. This process results in gold spheres roughly 17 nm in diameter that are coated with negatively charged citrate ions. The resulting particles are the seeds for the continuous growth of gold spheres.

### Synthesis of Dilution-Based Gold Spheres with Various Diameters

The dilution method was adopted and modified from a method developed by Bastús et al.^32^ A 95 mL solution of previously synthesized gold sphere seeds (∼17 nm) was used and mixed with 2 mL of a 60 mM sodium citrate solution. The mixture was dispersed in 53 mL of nanopure water to make a 150 mL growth solution. The growth solution was then heated to 90 °C and stabilized for 2 min. Then, 1 mL of a 25 mM HAuCl_4_ solution was added, and the mixture was left to react for 30 min. This process was repeated until the desired sizes of the gold spheres were achieved. To synthesize spheres roughly 30 nm in diameter, this process should be repeated 4 times, for 50 nm particles, repeat 7 times, and for 70 nm particles, repeat 9 times.

### Synthesis of Asymmetric Nanoparticles with Eccentric, Janus, and Tadpole Morphologies

To synthesize the gold-silica asymmetrically eccentric nanoparticle in Figure 1a, a growth solution was prepared by adding 3.8 mL of 2-propanol and 1.2 mL of nanopure water into a scintillation vial. Then, 160 μL of a 4.31 mM 4-MPAA ethanol solution and 160 μL of an aqueous 0.556 mM PAA_18_ solution ([4-MPAA]/[PAA_18_] = 7.78) were added dropwise to the vial with vigorous stirring. In parallel, a 2 mL solution of gold nanoparticles was centrifuged at 3700 g for 13 min, and the precipitate was then redispersed into 1 mL of ethanol. The resulting solution was added dropwise to the growth solution. The solution was then stirred for 1 h to allow ligands to exchange and redistribution on the surface of gold spheres. After one h, 350 μL of NH_4_OH solution (prepared by adding 40 μL of concentrated NH_4_OH into 1.5 mL of ethanol) was added to bring the pH of the solution to 10, and then 10 μL of 20%(v/v) TEOS in ethanol was added to form the silica shell. The reaction mixture was left for aging without stirring for 24 h. After the reaction, the solution was then purified by centrifuging at 3700 g for 13 min and redispersing in ethanol twice.

The above is a general procedure, and to fabricate other materials, the procedure was slightly modified. To study the PAA_18_ impact on the number of silica nucleation results in Figure 1, the amount of PAA_18_ solution is changed to reach the listed molar ratios in the paper. To change the level of eccentricity of silica coating results in Figure 2, the volume amount of the MPAA/PAA_18_ mixture with a fixed molar ratio of 7.78 was varied. More detailed experimental procedures are found in Results and Discussion Section.

To fabricate Janus nanostructures asymmetrically, 60 μL of a 4.31 mM 4-MPAA ethanol solution and 18 μL of an aqueous 5.56 mM PAA_18_ solution ([4-MPAA]/[PAA_18_] = 2.59) were used in the synthesis procedure (100 μL of ethanol and 142 μL of water were added to keep a consistent solvent ratio). After adding every agent, the reaction mixture was left stirring for 24 h before purification. To fabricate asymmetric tadpole nano/microstructures. For tadpole morphology, 200 μL of NH_4_OH solution was added to maintain a pH of 8 in the solution, and the aging time for the reaction mixture was extended to 120 h.

### Synthesis of Ternary Ag–Au–SiO_2_ and Core–shell Ag-Coated Au Nanostructures

To form ternary Ag on Au-SiO_2_ nanostructures, the 500 μL of as-synthesized Janus nanoparticles solution was washed with ethanol a few times and water one time via centrifugations (6200 rpm, 13 min) and finally redispersed in 100 μL of 11.11 mg/mL PVP (MW: 55,000 MW) solution. The solution was placed into the ThermoMixer and stirred at 700 rpm. During stirring, 200 μL of 10 mM HQ solution and 5 μL of 10 mM AgNO_3_ solution were added dropwise into the solution. After 2 h, Ag on Au-SiO_2_ structures were formed, and the solution was purified with water once via centrifugation (6200 rpm, 13 min) before further characterization or other use. To form core–shell nanostructures, a gold nanosphere solution (200 μL) instead of a Janus nanoparticle solution was used and redispersed into 100 μL, 11.11 mg/mL PVP (MW: 55,000 MW) solution. The remaining procedures are the same.

### Synthesis of Pt on Janus Au-SiO_2_ Nanostructures via Galvanic Replacement

After the initial purification of 100 μL of Ag–Au–SiO_2_ nanostructure solution, the precipitate was dispersed in an aqueous PVP solution (100 μL, 11.11 mg/mL, MW: 55,000 MW). Under stirring, 7.5 μL of 10 mM K_2_PtCl_4_ was added to the solution, and the mixture solution was stirred at 700 rpm and reacted for 1.5 h at room temperature. Then, the solution was centrifuged and washed with nanopure water once before the catalytic reduction of 4-NP and any characterization.

### Synthesis of Pt on Au Core–Shell Nanostructures

The purified 200 μL gold nanoparticle solution was mixed with 7.5 μL of 10 mM K_2_PtCl_4_ and 300 μL of 100 mM l-ascorbic acid. The solution was stirred at 700 rpm and reacted for 1.5 h at room temperature. Then, the solution was centrifuged and washed with nanopure water once before the catalytic reduction of 4-NP and any characterization.

### Catalytic Reduction of 4-Nitrophenol

The catalytic properties of Pt-based nanocatalysts were studied by in situ UV–vis monitoring of the change in absorption of 4-NP in the presence of NaBH_4_ at 400 nm. In a typical catalysis experiment, nanopure water (2.8 mL) was added to a quartz cuvette, followed by the addition of an aqueous solution of 4-NP (100 μL, 0.003 M) and the 5 times-diluted synthesized Pt-based nanocatalysts (100 μL). Upon beginning in situ monitoring, freshly prepared ice-cold NaBH_4_ (100 μL, 0.3 M) was added. The catalytic reduction was monitored by observing the change in absorption at a wavelength peak of 400 nm with a 1 min interval over a span of 30 min with the use of the UV–visible spectrophotometer.

### Liquid Crystal System Setup

#### Solution Preparation

Initially, 50 μL of 5CB was heated to 40 °C to ensure transition into the isotropic state, thereby improving miscibility. Subsequently, 35 μL of the nanoparticle dispersion was introduced into isotropic 5CB to form a homogeneous mixture. The prepared sample was then kept under quiescent conditions for 72 h, with the ethanol solvent allowed to evaporate slowly.

#### Surface Modification

All silicon wafers and glass substrates were first cleaned by using a piranha solution consisting of sulfuric acid and hydrogen peroxide mixed in a 3:1 ratio. The substrates were immersed in this solution at 130 °C for 1 h, followed by thorough rinsing with deionized water at least 3 times to eliminate the residual contaminants. Homeotropic alignment layers were prepared by immersing the glass slides in a solution of octadecyltrichlorosilane (13.8 μL) dissolved in 120 mL of heptane for 1 h. The treated substrates were subsequently rinsed twice with dichloromethane and dried under a stream of nitrogen. For planar alignment, a 0.05 wt % PMMAZO solution in chlorobenzene was spin-coated onto silicon wafers, which were then placed in a nitrogen atmosphere and thermally annealed at 250 °C for 5 min.

#### Cell Construction

Silicon wafers and glass slides were cut to the desired dimensions and cleaned by using a stream of nitrogen gas. A Mylar film spacer was employed to define a uniform cell gap of 1.5 μm for the liquid crystal confinement. Planar alignment cells were assembled by using piranha-cleaned silicon wafers paired with piranha-treated glass slides. Hybrid alignment cells consisted of a piranha-treated silicon wafer as the bottom substrate and an OTS-functionalized glass slide as the top substrate. The cells were constructed by using a PMMAZO-coated silicon wafer in combination with an OTS-treated glass slide. All cell assemblies were sealed using an epoxy adhesive. To achieve uniform nanoparticle dispersion, the suspension was subjected to ultrasonication for 90 min, which effectively reduced particle agglomeration and promoted a homogeneous distribution. The mixture was subsequently heated to 40 °C and vortex-mixed for 5 min, a process repeated three times to further improve dispersion within 5CB. The solution was then reheated to 40 °C, and approximately, 3.5 μL of the mixture was injected into the cells, which were filled via capillary action.

### Instruments and Measurement

Scanning transmission electron microscopy (STEM) imaging was conducted using a JEOL-7200F field emission SEM operated at 30 kV. All of the material sizes were measured via STEM images using the ImageJ software. The STEM samples were prepared by drop-casting samples onto copper grids. UV–vis absorption spectra were measured by using a Jasco V 670 UV–vis-NIR spectrophotometer. Images of the LC cells were obtained by polarization microscopy (BX53 Olympus). Periods of the striped pattern and sizes of fingerprint domains were measured by the image processing program (ImageJ).

## Supplementary Material



## Notes

STEM image and size histogram of synthesized gold nanoparticles; additional STEM image of silica-coated GNPs with various synthetic conditions; STEM image of Pt–Au core–shell nanostructure; and time-dependent UV spectra of 4-nitrophenol by NaBH_4_ and catalyzed by the core–shell nanostructure (PDF)
