# Supplementary material for: Asymmetric Silica–Gold Nano/Microparticles: Eccentric, Janus, Tadpole Structures, and Their Applications
Source: ACS Appl Mater Interfaces. 2026 Apr 13;18(16):23815–26. doi: 10.1021/acsami.6c02756 (PMC13133778; doi:10.1021/acsami.6c02756)
Supplement: Supplementary file 1 [file am6c02756_si_001.pdf]

Supporting Information

Asymmetric Silica–Gold Nano/Microparticles: Eccentric, Janus, Tadpole Structures and Their Applications

Teagan Hamlett [a§], Xiaowei Wang [b§], Jaden Poellot [a], Joseph E. Doebler [a], Sid Hashemi [b], Xiao Li [b], Ying Bao [a*]*

[a] Department of Chemistry, Western Washington University, Bellingham, WA 98226, United States

[b] Materials Science and Engineering Department, University of North Texas, Denton, TX 76207, United States

*Email: Ying.Bao@wwu.edu; Xiao.Li@unt.edu

[§] T. H. and X. W. contributed equally to this work

SI Figure lists:

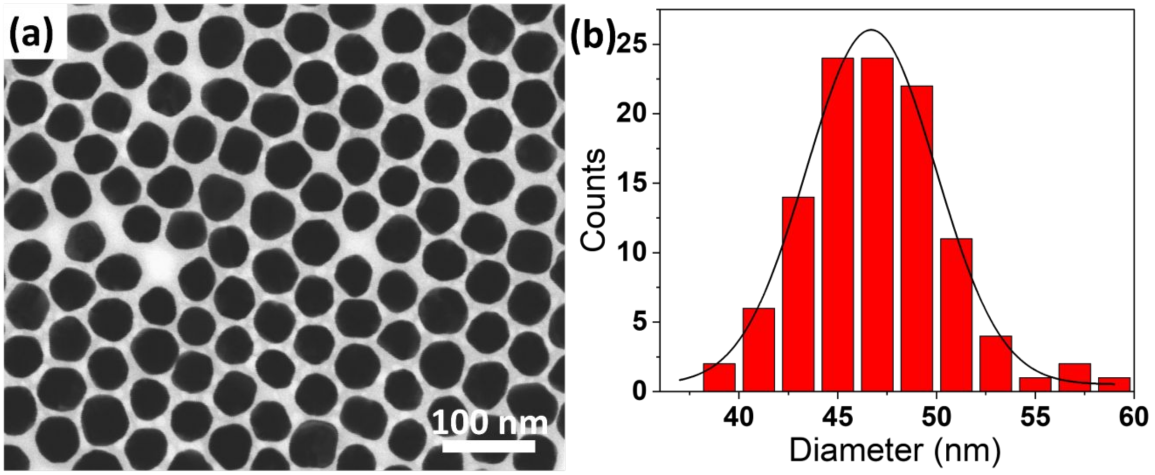

Figure S1. (a) STEM image and (b) size histogram of synthesized citrate coated gold nanoparticles based on a modified method by Neus G Bastús et. al.[1] .

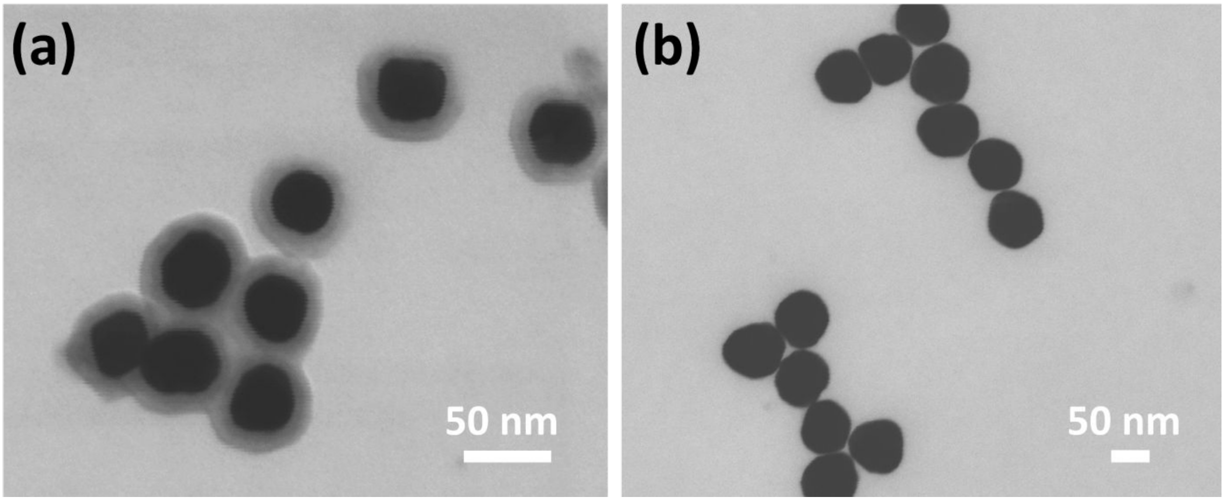

Figure S2. STEM images of silica deposited GNPs with (a) only 4-MPAA ligands, (b) only PAA_{18} ligand and with MPAA:PAA ratio of 7.78.

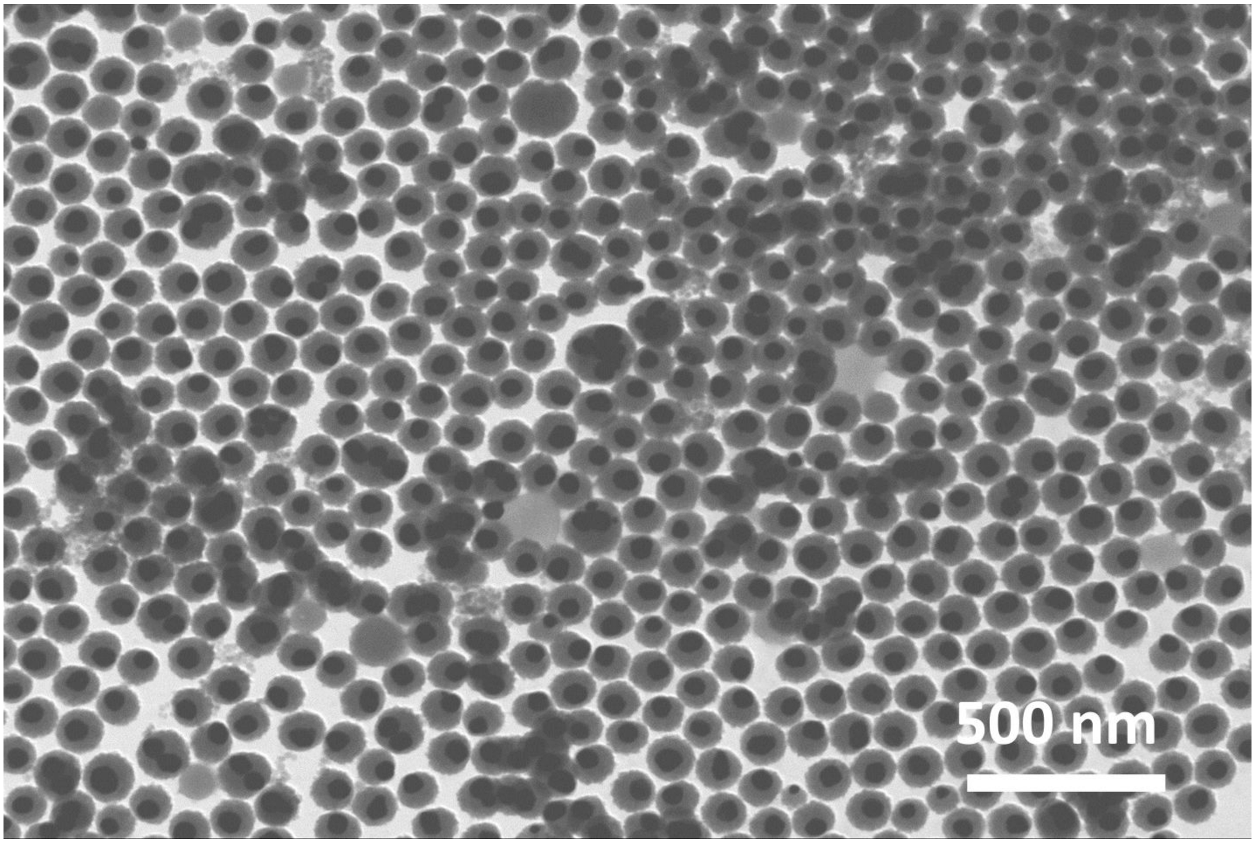

Figure S3. An low magnification STEM image of silica deposited GNPs using MPAA:PAA_{18} ratio of 7.78.

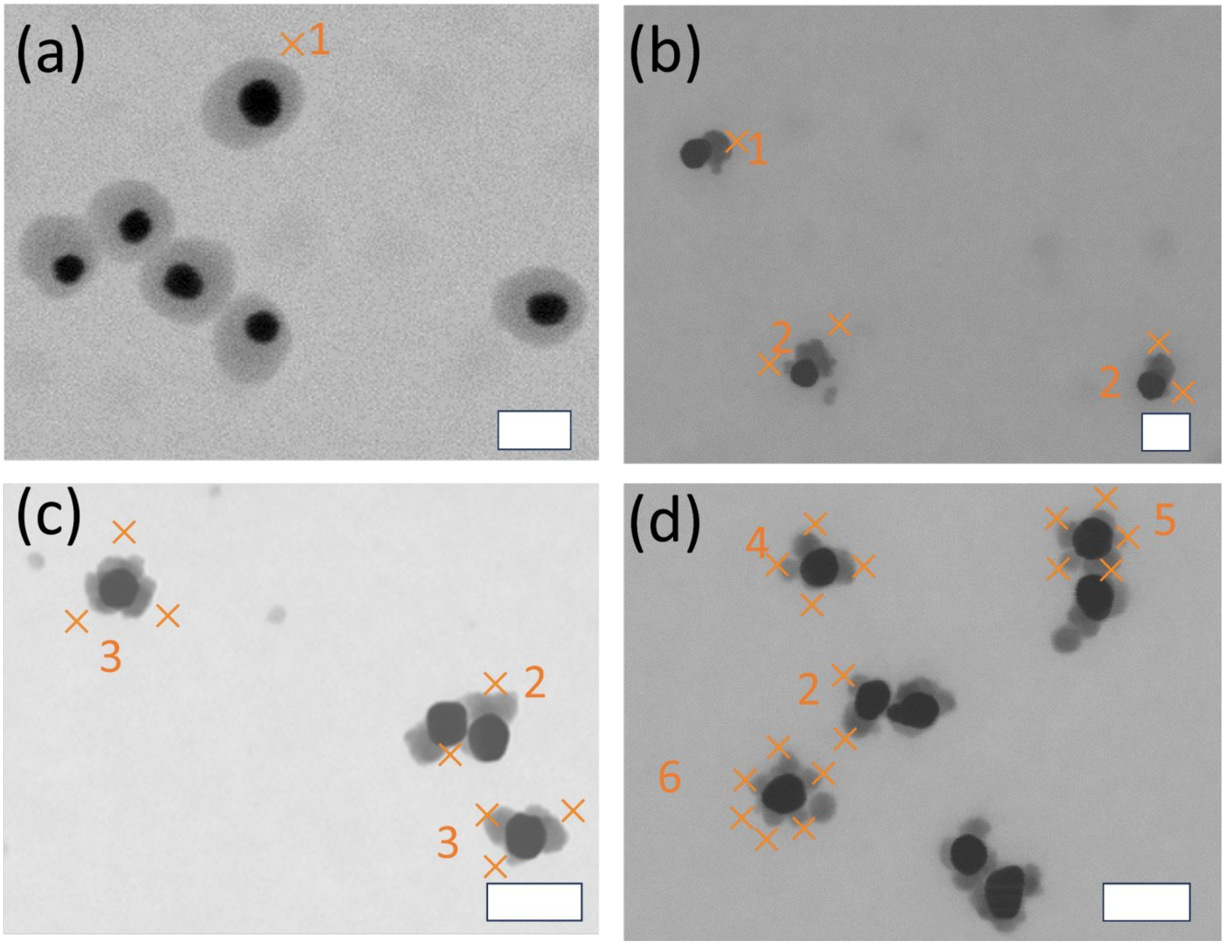

Figure S4. Examples on counting maximum number of domains in each particle on Figure 2(a-d). Scale bar: 100 nm.

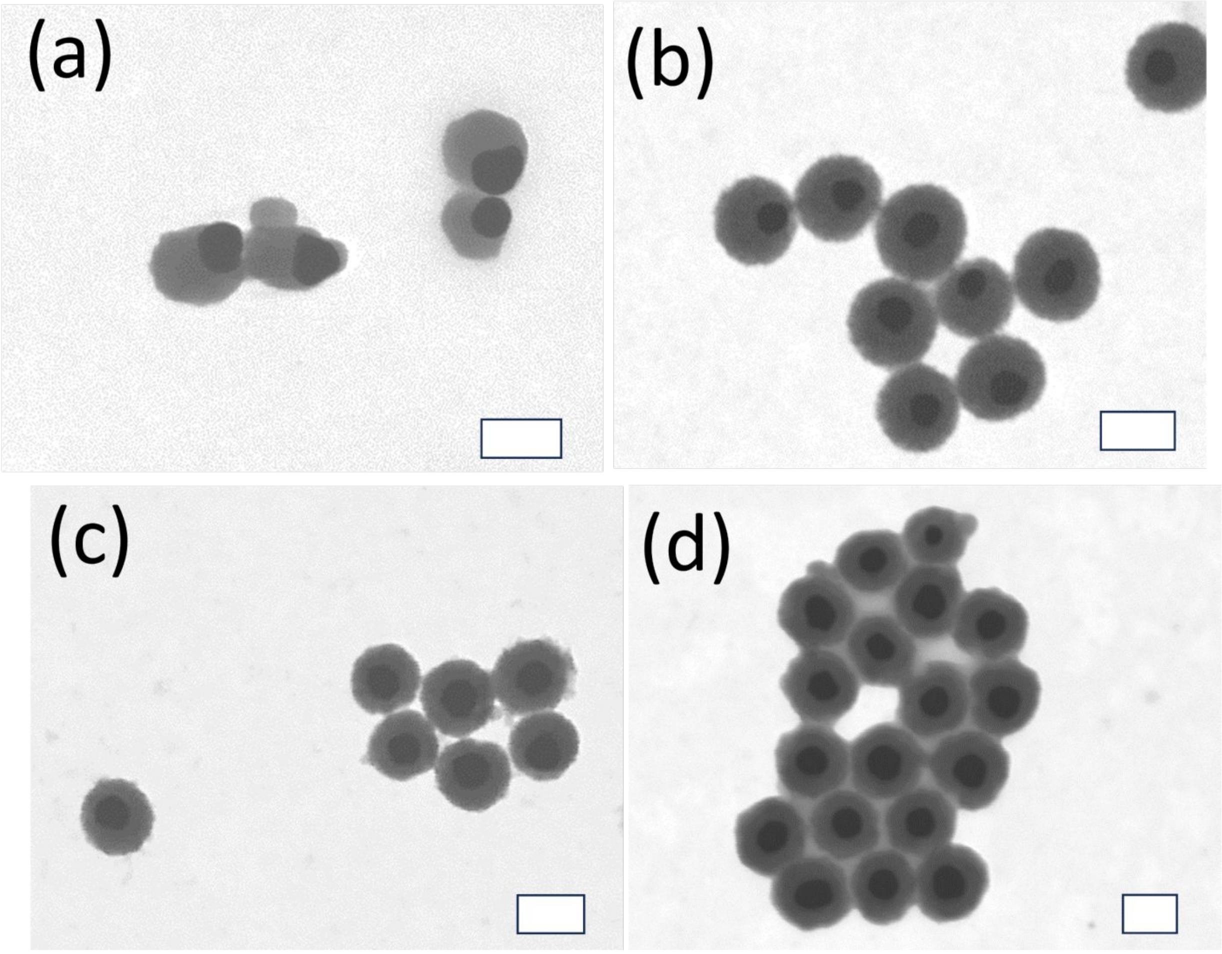

Figure S5. (a-b) STEM images of Janus eccentric growths using varying concentration of the 4-MPAA ligands with (a) 0.039 mM (b) 0.21 mM (c) 0.41 mM (d) 0.62 mM. Scale bar: 100 nm.

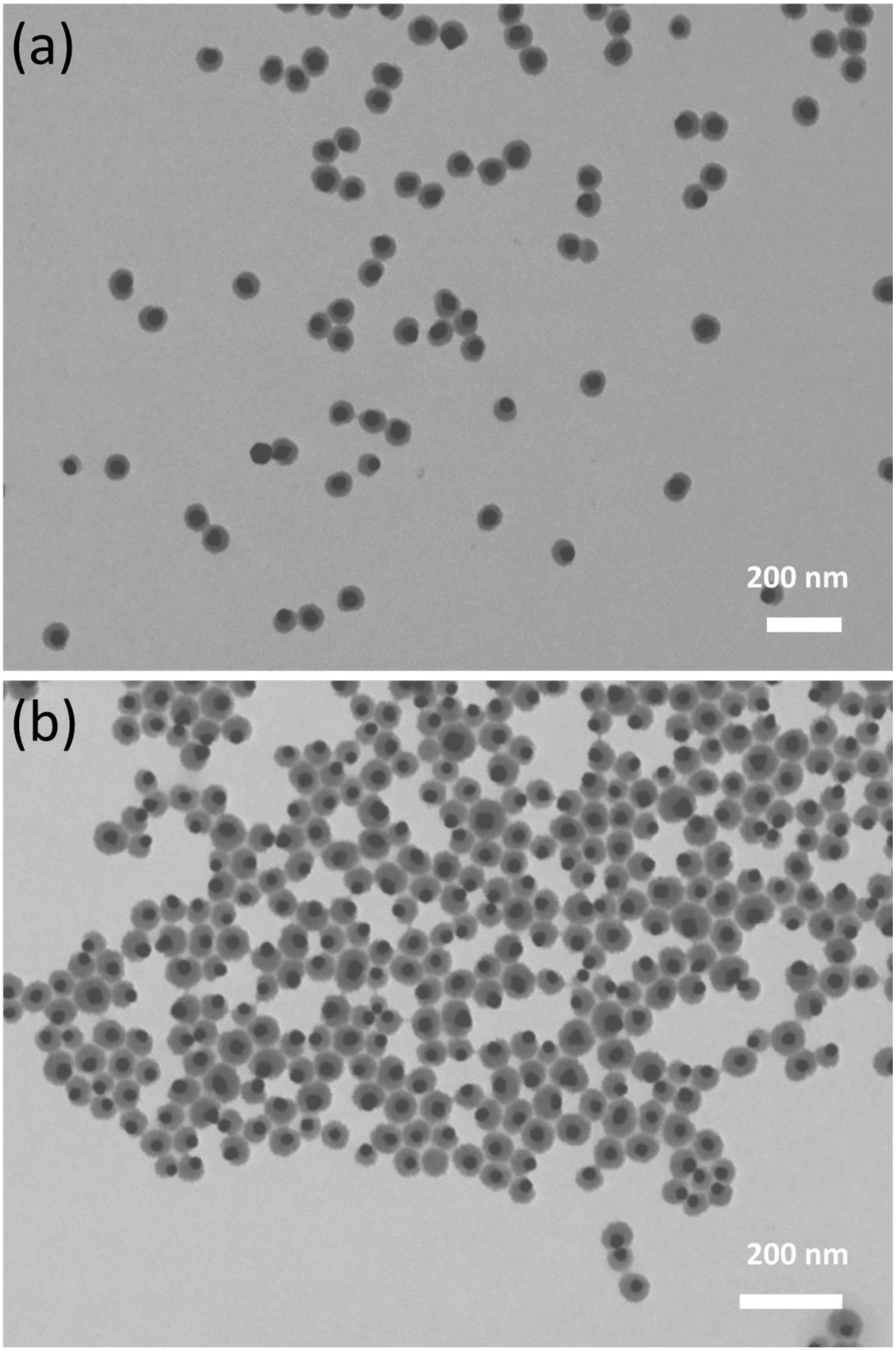

Figure S6: Low magnification STEM images of Au-SiO_2 Janus nanostructures using [4-MPAA]/[PAA]=2.59 while 0.039 mM of 4-MPAA concentration using GNP core size with (a) 50 nm and (b) 30 nm.

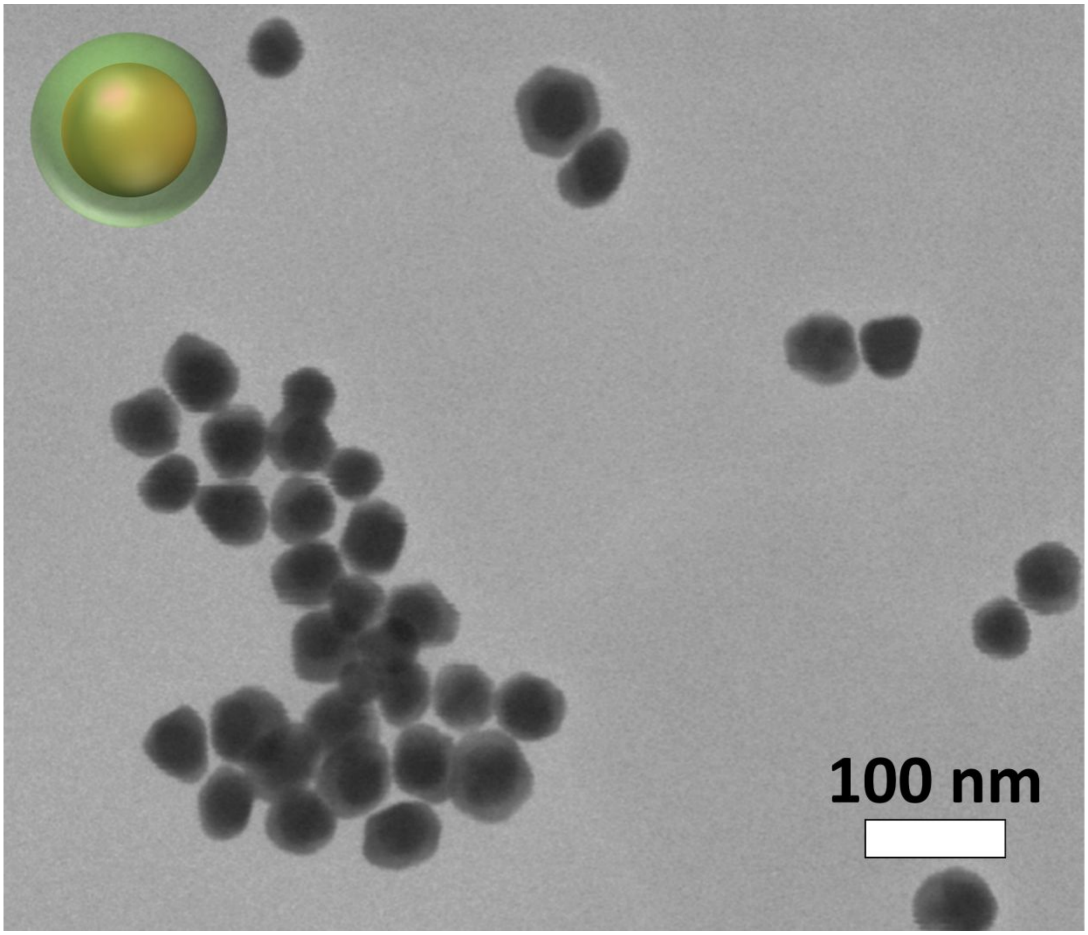

Figure S7: STEM images of Ag-Au core shell nanostructure obtained by using purified citrate coated GNP as seed and deposited silver on the surface.

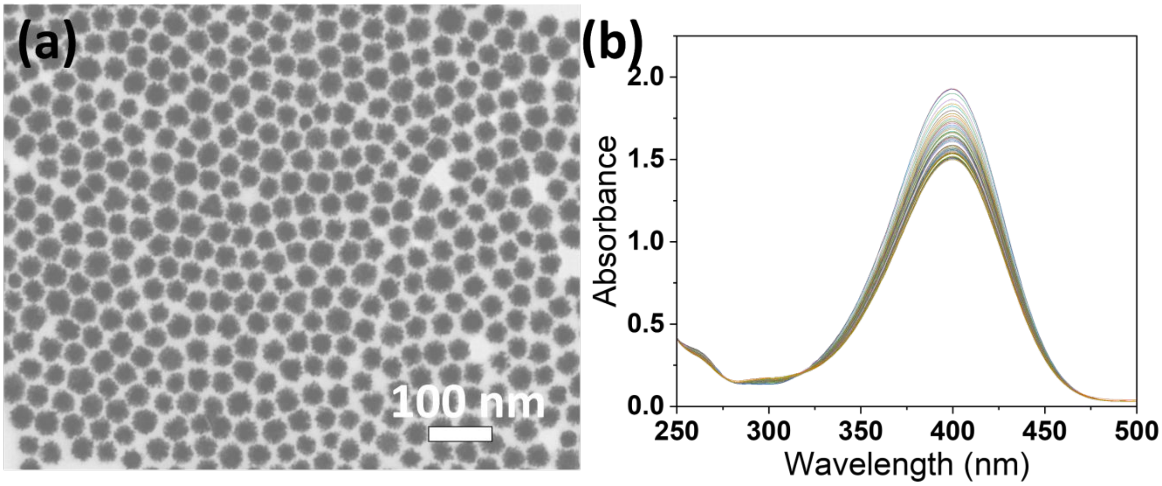

Figure S8: (a) STEM image of Pt-Au core-shell nanostructures; (b) Time dependent UV-vis absorption spectra of 4-NP reduced by $NaBH_4$ and catalyzed by the Pt-Au core-shell nanostructures. Scale bar: 100 nm.

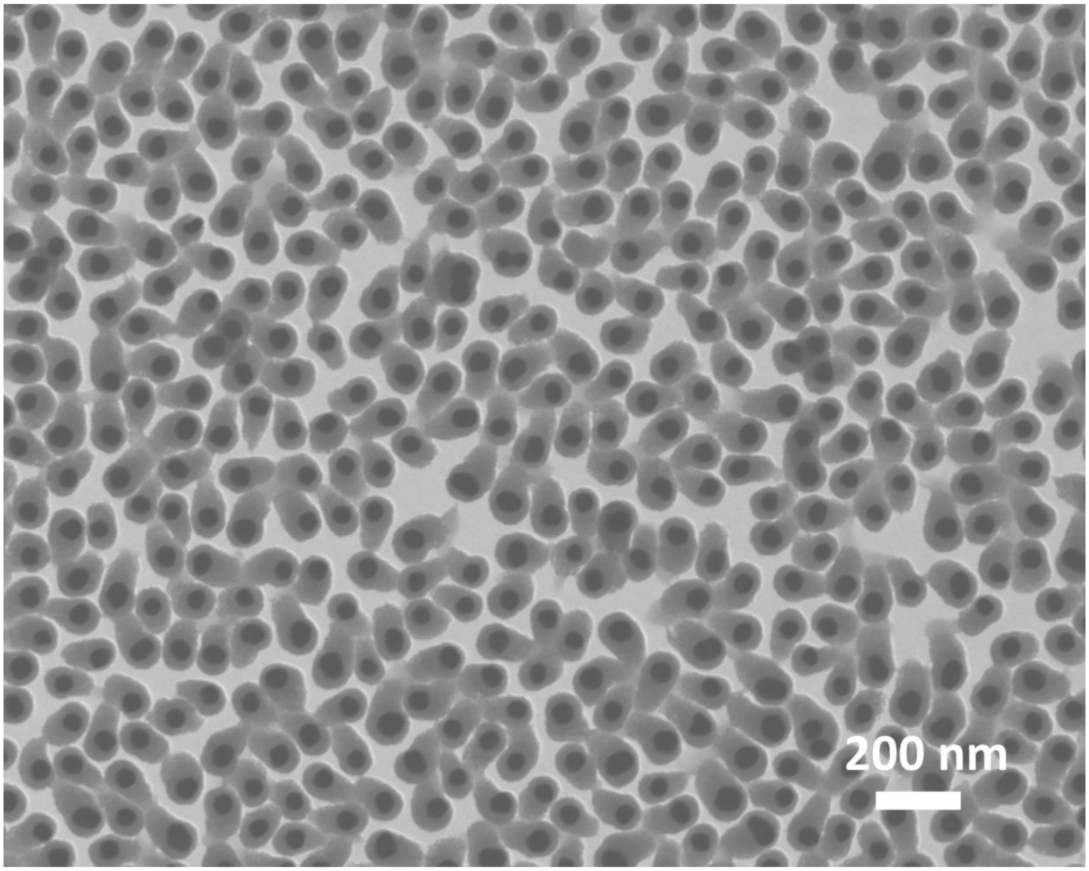

Figure S9. STEM image of asymmetric tadpole structure formed under pH 8, and 16 μL of 4-MPAA and PAA18 with [4-MPAA]/[PAA18]=7.78.

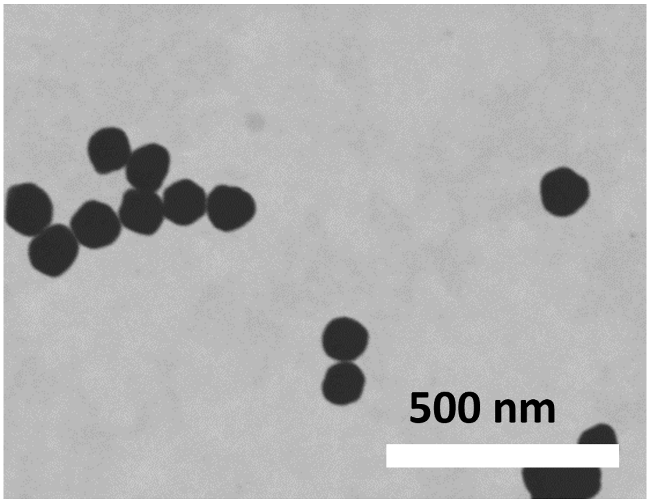

Figure S10. STEM image of resulted structure formed under pH 7, and 16 μL of 4-MPAA and PAA_{18} with [4-MPAA]/[PAA_{18}]=7.78.

1. Bastús, N. G.; Comenge, J.; Puntes, V., Kinetically Controlled Seeded Growth Synthesis of Citrate-Stabilized Gold Nanoparticles of up to 200 nm: Size Focusing versus Ostwald Ripening. *Langmuir* **2011,** *27* (17), 11098-11105.
